# Predictors of fifty days in-hospital mortality in patients with culture negative neutrocytic ascites

**DOI:** 10.1186/s12876-017-0621-x

**Published:** 2017-05-16

**Authors:** Chinmaya Kumar Bal, Vikram Bhatia, Ripu Daman

**Affiliations:** 1 Department of Hepatology, Institute of Liver and Biliary Sciences (ILBS), New Delhi, India; 2 Institute of Liver and Biliary Sciences (ILBS), New Delhi, India

**Keywords:** Culture negative neutrocytic ascites, Spontaneous bacterial peritonitis, Liver cirrhosis, Mortality rate, Acute kidney injury, Septic shock, Model for end stage liver disease, Aspartate transaminase, Alanine transaminase

## Abstract

**Background:**

Culture negative neutrocytic ascites is a variant of spontaneous bacterial peritonitis. But there are conflicting reports regarding the mortality associated with culture negativeneutrocytic ascites. Therefore we aim to determine the predictors of mortality associated with culture negativeneutrocytic ascites in a larger sample population.

**Methods:**

We analysed 170 patients consecutively admitted to intensive care unit with diagnosis of culture negative neutrocytic ascites. The clinical, laboratory parameters, etiology of liver cirrhosis was determined along with the scores like model for end stage liver disease, child turcotte pugh were recorded.

**Results:**

The 50 day in-hospital mortality rate in culture negative neutrocytic ascites was 39.41% (*n* = 67). In univariate analysis, means of parameters like total leucocyte count, urea, bilirubin, alanine transaminase, aspartate transaminase, international normalized ratio, acute kidney injury, septic shock, hepatic encephalopathy and model for end stage liver disease were significantly different among survived and those who died (*P* value ≤0.05). Cox proportional regression model showed the hazard ratio (HR) of acute kidney injury was 2.212 (95% CI: 1.334–3.667), septic shock (HR = 1.895, 95% CI: 1.081–3.323) and model for end stage liver disease (HR = 1.054, 95% CI: 1.020–1.090). Receiver operating characteristics curve showed aspartate aminotransferase (AST) had highest area under the curve 0.761 (95% CI: 0.625–0.785).

**Conclusion:**

Patients with culture negative neutrocytic ascites have a mortality rate comparable to spontaneous bacterial peritonitis. aspartate aminotransferase, alanine aminotransferase (ALT), acute kidney injury (AKI), model for end stage liver disease (MELD) and septic shock are the independent predictors of 50 days in-hospital mortality in culture negative neutrocytic ascites.

## Background

Spontaneous bacterial peritonitis is a (SBP) common presentation of ascitic fluid infection in patients with advanced liver cirrhosis. There are three variants of spontaneous bacterial peritonitis. Culture negative neutrocytic ascites, monomicrobialbacterascites, polymicrobialbacterascites. The term culture-negative neutrocytic ascites (CNNA) was proposed in 1984 by Runyon [1].The diagnosis was made with 1) elevated ascitic fluid polymorponuclear cell count ≥250 cells/mm^3^ 2) negative ascitic fluid culture 3) absence of antibiotic therapy in the last one month 4) no evidence of surgically treatable intraabdominal source of infection or pancreatitis.

There are conflicting reports regarding the natural history and the mortality associated with culture-negative neutrocytic ascites. Pelletier et al. and Saleh et al., reported spontaneous resolution of culture-negative neutrocytic ascites along with lower mortality compared to spontaneous bacterial peritonitis [2, 3].While Runyon and Hoefs advocated comparable mortality rates among culture-negative neutrocytic ascites and spontaneous bacterial peritonitis [1]. Both the study were based on small sample size (*n* = 15),also there is no study on the predictors of the mortality in culture negative neutrocytic ascites. Hence we aim to study the clinical course and prognosis associated with culture negative neutrocytic ascites with a larger sample population.

## Methods

The diagnosis of culture negative neutrocytic ascites was made by presence of ascitic PMN (poly-morphonuclear cell) count ≥250 cells/mm^3^ in the ascitic fluid in the absence of culture positivity [1]. *Exclusion criteria*: a) Patient with cirrhosis and ascitic fluid absolute polymorphonuclear leukocyte (PMN) count ≤250 cells/mm^3^. b) Positive ascitic fluid culture or surgically treatable intraabdominal source of infection or pancreatitis. c) Patients presented with ascites not related to liver cirrhosis d) Patients with advanced malignancy, tuberculosis, immunodeficiency, traumatic paracentesis e) Patients exposed to systemic antibiotics within last one month. f) Patients with multiple episodes of ascites.

A total number of one hundred and seventy patients were consecutively registered that were admitted to the hepatology intensive care unit of Institute of Liver and Biliary Sciences, New Delhi between June 2013 and June 2014. The study was approved by Institutional Ethics Committee. The consent was obtained from all patients. The guidelines of Helsinki Declaration were followed [4].

The clinical, laboratory parameters, other comorbid condition and the etiology of cirrhosis were determined at the admission to the intensive care unit (Table 1). As a protocol all the patients admitted to intensive care unit with ascites due to liver cirrhosis, underwent diagnostic paracentesis under aseptic precautions within 24 h of admission in the absence of severe coagulopathy. Response tap was done after 48 h. Our entire culture-negative neutrocytic ascites patients received parenteral aminoglycosides with clavulanic acid or third/fourth generation cephalosporin. 10 ml bottle was used for ascitic fluid analysis which includes ascitic fluid protein, albumin, total leukocyte count, differential count and polymorphonuclear cell count [5].The patient demographics like age, gender along with clinical features like fever, abdominal pain, hepatic encephalopathy, upper gastro intestinal bleed and other laboratory data was collected. Similarly blood cultures were also drawn for aerobic and anaerobic cultures before starting antibiotics. Acute kidney injury was defined by AKIN criteria [6]. Acute kidney injury was managed by intravenous albumin and terlipressin infusion. Previous medication histories along with exposure to complementary and alternative medications were reviewed while considering the development of ascites and worsening of kidney function. NSAIDs, diuretics and nephrotoxic drugs were stopped after development of acute kidney injury. The dose was titrated as per response and tolerance. American College of Chest Physicians/Society of Critical Care Medicine consensus conference criteria were used to diagnose septic shock [7].

**Table 1:** Baseline characteristics of the hospitalized patients with culture negative neutrocytic ascites in cirrhosis

Variables	Overall (*n* = 170)	Survivors (*n* = 103)	Deaths (*n* = 67)	*P* value
Demographic data	50.59 ± 12.39	50.43 ± 13.02	50.84 ± 11.45	0.834
Age (yr) mean ± SD	137 (80.58)	82 (79.61)	55 (82.08)	0.699
Male (%)	12.23 ± 10.25	10.80 ± 8.10	14.43 ± 12..63	0.023
Stay duration (days) mean ± SD				
Etiology of cirrhosis (%)				
Ethanol	79 (46.47)	41 (39.80)	38 (56.71)	0.345
Cryptogenic	40 (23.52)	25 (24.27)	15 (22.38)	0.221
Hepatitic C virus	19 (11.17)	15 (14.56)	4 (5.97)	0.098
Hepatitis B virus	14 (8.23)	10 (9.70)	4 (5.97)	0.114
Non Alcoholic Fatty Liver Disease	10 (5.88)	6 (5.82)	4 (5.97)	0.179
Clinical data (%)				
Diabetes	38 (22.35)	22 (21.35)	16 (23.88)	0.71
Acute Kidney Injury	70 (41.17)	27 (26.21)	43 (64.17)	≤0.001
Respiratory failure	8 (4.80)	5 (4.85)	3 (4.47)	0.91
Hepatic encephalopathy	85 (50.00)	42 (40.77)	43 (64.17)	0.003
Septic Shock	20 (11.76)	02 (1.94)	18 (26.86)	≤0.001
Laboratory data (mean ± SD)				
Hemoglobin (g/dL)	9.46 ± 1.89	9.60 ± 1.74	9.26 ± 2.10	0.255
Total leucocyte count (10^3^/μL	12.92 ± 8.09	11.44 ± 7.37	15.16 ± 8.65	0.003
Platelet count (mmol/L)	129.02 ± 106.48	136.25 ± 111.21	118.13 ± 98.72	0.281
Sodium (mEq/L)	131.97 ± 7.73	132.52 ± 6.61	131.13 ± 9.18	0.251
Urea (mg/dL)	70.14 ± 53.26	62.52 ± 48.20	81.87 ± 58.66	0.021 0.365
Creatinine (mg/dL)	1.63 ± 1.31	1.56 ± 1.35	1.75 ± 1.23	
Bilirubin (mg/dL)	7.81 ± 8.68	5.77 ± 6.37	10.96 ± 10.65	≤0.001
Aspartate Transaminase (U/L)	113.38 ± ±221.81	71.25 ± 70.20	178.13 ± 333.68	0.002
Alanine Transaminase (U/L)	57.22 ± 104.00	39.59 ± 33.00	84.33 ± 157.42	0.006
INR	2.29 ± 1.14	2.08 ± 1.14	2.61 ± 1.07	0.003
Albumin (g/dL)	2.31 ± 0.48	2.31 ± 0.46	2.31 ± 0.51	0.981
Ascitic fluid leucocyte count (cells/mm^3^)	4285.65 ± 5616.71	4770.63 ± 5745.64	3540.07 ± 5369.37	0.163
Ascitic fluid PMN (cells/mm^3^)	3554.70 ± 5083.02	3960.09 ± 5215.57	2931.49 ± 4844.38	0.198
Scores (mean ± SD)				
Child Turcotte Pugh (B/C)	10.68 ± 1.80	10.47 ± 1.93	11.01 ± 1.53	0.052
MELD	24.36 ± 7.99	22.08 ± 7.12	27.88 ± 8.03	≤0.001
MELD-sodium	27.29 ± 7.40	25.20 ± 7.14	30.49 ± 6.64	≤0.001

*Abbreviations*: *INR* International Normalized Ratio, *MELD* Model for End stage Liver Disease

Statistical analysis was performed using SPSS version 20.0 for windows. All the variables were assumed to be normally distributed with equal variance. The continuous variables were explained as mean ± standard deviation (SD). Categorical variables were explained as proportions. The means of continuous variables were compared using student’s *t* test. The means of categorical variables were compared using chi square and fisher exact test. Multivariate logistic regression was used to analyse the statistically significant variable. Cox proportional regression model was used to estimate the hazard rates of the statistical significant variable, adjusted by age and gender. All the *P*-values were two sided and considered a statistically significant if ≤ 0.05. The desired power (type II error) is set at 0.8.

The predictive accuracy of prognostic variables like acute kidney injury, model for end stage liver disease, septic shock, aspartate aminotransferase and alanine aminotransferase were drawn using receiver operating characteristics (ROC) curves. The area under the curve (AUC) with 95% confidence interval was with significance level was deducted from receiver operating characteristics curve. Kaplan Meier survival analysis was used for the 50 day in hospital survival analysis. The best cut-off point for the continuous predictors like model for end stage liver disease, aspartate aminotransferase, alanine aminotransferase using acceptable sensitivity and specificity in the receiver operating characteristics analysis were used for 50 day in hospital survival analysis. The categorical predictors like acute kidney injury and septic shock also used in survival analysis.

## Results

Total of 170 culture negative neutrocytic ascites patients with decompensated cirrhosis were included in the study. Culture negative neutrocytic ascites were first episode for the all the patients. We designed dedicated research protocol with target oriented questionnaires to include only first episode of ascites in our study. The 50 day in-hospital mortality rate was 39.41% (*n* = 67). The median for non survivors were 14 days, with 100% of all the deaths (*n* = 67) occurring within 50 days of intensive care unit (ICU) admission, hence we decided to calculate the predictive powers of different prognostic parameters for 50 days in hospital mortality rates among culture negative neutrocytic ascites patients. In univariate analysis, means of blood parameters like total leucocyte count, urea, bilirubin, alanine aminotransferase, aspartate aminotransferase, international normalized ratio (INR) were significantly different among survived and those who died. The differences in other clinical parameters like septic shock, acute kidney injury, model for end stage liver disease and hepatic encephalopathy between two groups were also statistically significant. The means with standard deviation of ascitic fluid polymorphonuclear cell count among died vs survived was 2931.49 ± 4844.38 vs 3960.09 ± 5215.57. Surprisingly ascitic fluid total leucocyte count and poly morphonuclear cell count were not significantly different between the groups (Table 1). The baseline characteristics of the hospitalized patients with culture negative neutrocytic ascites is shown in Table 1. Mean age was 50.59 ± 12.39 with male predominance (80.6%). Ethanol consumption was the predominant cause of liver cirrhosis (46.5%) followed by cryptogenic (23.5%). Hepatitis C virus constitutes only 11% in our study. Blood total leucocyte count is higher among those died vs survived (15.16 ± 8.65 vs 11.44 ± 7.34) but it does not correspond to ascitic fluid total leucocyte count or polymorphonuclear cell count level. Bilirubin, urea, INR level was higher among who died compared to the survivors. Total of 50% (*n* = 85) had hepatic encephalopathy with 64.2% deaths (*n* = 43, *p* = 0.003). Overall 41.2% (*n* = 70) had AKI among hospitalized patients out of which 64.2% died (*n* = 43, *P* ≤ 0.001). The non survivors had a higher proportion of septic shock compared with survivors (26.86% vs 1.94%), *P* ≤ 0.001. The non survivors had high MELD score comparing to the survivors (27.88 ± 8.03 vs 22.08 ± 7.12), *P* ≤ 0.001. Child-Turcotte-Pugh (CTP) (B/C) scores were comparable among the groups. The mean CTP scores were 10.68 (SD ± 1.82).

Cox proportional regression model showed AKI (HR = 2.212, 95% CI: 1.334–3.667), septic shock (HR = 1.895, 95% CI: 1.081–3.323) and MELD (HR = 1.054, 95% CI: 1.020–1.090) (Table 2). Interestingly on multivariate analysis aspartate aminotransferase (*p* = 0.018), alanine aminotransferase (*p* = 0.005), bilirubin (*p* = 0.001) international normalized ratio (*p* = 0.001) were found to be independent predictors of 50 day in-hospital mortality associated with culture negative neutrocytic ascites. We did not include other variables like total leukocyte count, serum bilirubin, INR since these were already incorporated in MELD-Na and septic shock.

**Table 2:** Cox proportional regression analysis of risk factors for culture negative neutrocytic ascites related in-hospital related mortality

Variables	^a^HR (95% CI)	*P* value
Acute Kidney Injury	2.212 (1.334–3.667)	0.002
Hepatic encephalopathy	1.523 (0.917–2.529)	0.104
Septic Shcok	1.895 (1.081–3.323)	0.026
Total leucocyte count	1.030 (1.002–1.059)	0.037
Urea	1.003 (0.999–1.007)	0.163
Bilirubin	1.041 (1.016–1.066)	0.001
Aspartate Transaminase	1.001 (1.000–1.001)	0.018
Alanine Transaminase	1.002 (1.001–1.004)	0.005
INR	1.208 (1.012–1.441)	0.036
MELD	1.054 (1.020–1.090)	0.002

*Abbreviations*: *INR* International Normalized ratio, *MELD* Model for End stage Liver Disease

^a^Hazard ratio (HR) adjusted for age and gender

**Fig. 1:**
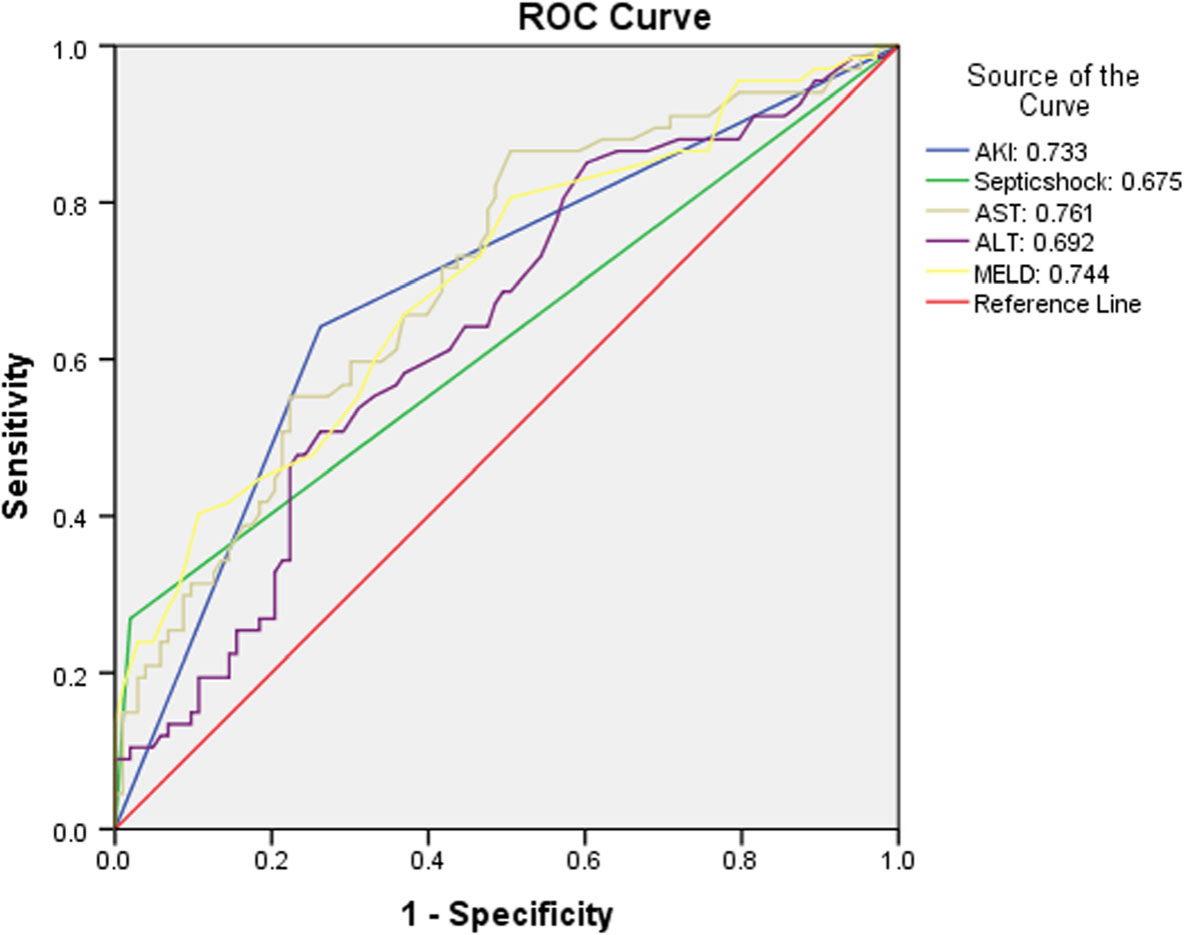
Receiver Operating Characteristics curve of prognostic variables in culture negative neutrocytic ascites. *AKI*: Acute Kidney Injury, *AST*: Aspartate Transaminase, *ALT*: Alanine Transaminase, *MELD*: Model for End stage Liver Disease

**Fig. 2:**
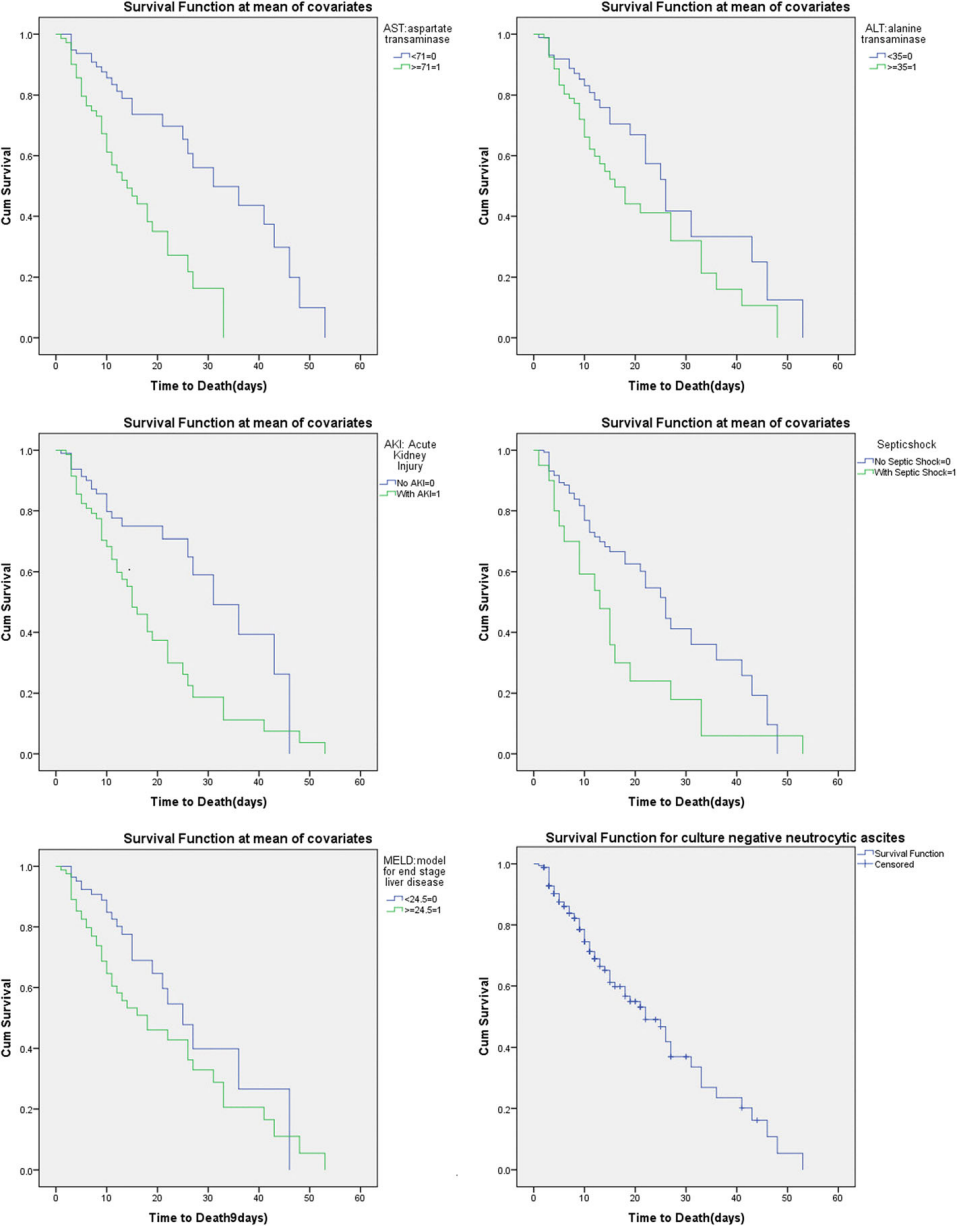
Kaplan Meier survival analysis for the 50 days survival in culture negative neutrocytic ascites

Receiver operating characteristics curve for acute kidney injury, septic shock and model for end stage liver disease, aspartate aminotransferase, alanine aminotransferase are shown in Fig. 1. Aspartate aminotransferase had highest AUC 0.761 (95% CI: 0.625–0.785), followed by mode for end stage liver disease (AUC: 0.744, 95% CI: 0.620–0.782), acute kidney injury (AUC: 0.733, 95% CI: 0.607–0.773), alanine aminotransferase (AUC: 0.692, 95% CI: 0.555–0.724), septic shock (AUC: 0.675, 95% CI: 0.535–0.795) (Table 3). The Kaplan Meier survival analysis curve was plotted for 50 day survival period with individual prognostic variables like acute kidney injury, model for end stage liver disease, septic shock, aspartate aminotransferase, alanine aminotransferase. (Fig. 2) The cut off value for MELD derived from receiver operating characteristics curves with best ability to predict 50 day in-hospital mortality in culture negative neutrocytic ascites was 24.5 with sensitivity and specificity 63%, 66% respectively. The cut off for aspartate aminotransferase was 71 with sensitivity and specificity 60%, 70% respectively.

**Table 3:** Diagnostic accuracy of prognostic variables to predict culture negative neutrocytic ascites related in-hospital mortality

Predictors	^a^AUC (95% CI)	*P* value
Acute Kidney injury	0.733 (0.607–0.773)	≤0.001
Septic Shock	0.675 (0.535–0.795)	0.006
Aspartate Transaminase	0.761 (0.625–0.785)	≤0.001
Alanine Transaminase	0.692 (0.555–0.724)	0.002
MELD	0.744 (0.620–0.782)	≤0.001

^a^Area Under Curve (AUC). MELD: Model for End stage Liver Disease

## Discussion

Ascitic fluid infection is a common presentation in liver cirrhosis. Culture negative neutrocytic ascites is considered as a variant of spontaneous bacterial peritonitis [1 ].Our study reports 39.4% mortality rate in culture-negative neutrocytic ascites . The whole idea of using known prognostic parameters is to validate our study which agrees with the already established facts that septic shock, model for end stage liver disease, CTP, acute kidney injury are independent prognostic factors in liver cirrhosis. We tried to prognosticate all the clinically relevant variables across the board while comparing the survivors vs. non survivors. We found hepatic encephalopathy and CTP, though a clinically relevant and statistically significant among the groups, it has poor predictive ability as far as mortality is concerned. Moreover aspartate aminotransferase, alanine aminotransferase have significant predictive ability which is against the conventional wisdom. In this study aspartate aminotransferase has the highest independent predictive ability of 50 day in hospital mortality rate with AUC 0.761 (95% CI: 0.625–0.785, *p* ≤0.001). Other statistical significant predictors in the descending order of predictive ability include model for end stage liver disease (AUC: 0.744), acute kidney injury (AUC: 0.733), alanine aminoransaminase (AUC: 0.692) and septic shock (AUC: 0.675).

The hazards of mortality was highest for acute kidney injury with HR 2.212 (95% CI: 1.334–3.667, *p* ≤ 0.002) followed by septic shock (HR: 1.895), coagulopathy (HR: 1.208), and model for end stage liver disease (HR: 1.054). One of the clinically relevant indicators which were not statistically significant includes hepatic encephalopathy. Kaplan Meier survival analysis showed aspartate aminotransferase, alanine aminotransferase, acute kidney injury, model for end stage liver disease and septic shock as distinct independent predictors of 50 day in-hospital mortality associated with culture-negative neutrocytic ascites . This stratification will further improve the quality of care of hospitalized patients with CNNA leading to reduction of short term mortality. The cut off value for model for end stage liver disease (24.5), aspartate aminotransferase (71) and alanine aminotransferase (35) can be applied to triage the high risk patients upon blood and laboratory evaluation after admission into intensive care. Our results clearly depict statistical significance of AST. It is worth to mention here that this finding is also clinically relevant. This is hard to believe as the results are against conventional wisdom (AKI,MELD and Septic Shock) It took us by surprise as none of the current studies to our knowledge elucidates this fact. The sensitivity and specificity of AST is low (60% and 70% respectively). It should be kept in mind that this value is correlated to the cut off value of 71. If we decrease the cut off value sensitivity will increase, specificity will decrease and vice versa if we increase the cut off value. A low cut cut off value mean more false positives. A high cut off will increase the specificity but we might miss many clinically significant cases due to more number of true negatives. Hence we balanced this critical situation with an acceptable sensitivity and specificity values which can be used for screening procedures in clinical cases.

It is important to mention that CTP and ascitic fluid polymorphonuclear count scores are not statistical significant predictors of mortality in culture negative neutrocytic ascites. Our results of culture negativity was based on the first negative culture results with in 24 h of hospital admission. We don’t know how many become culture positive after that. It is not clear those become culture positive after initial negative results should be categorised into SBP or CNNA.We don’t know the recurrence rate or conversation rate of culture negative neutrocytic ascites.

Culture negative neutrocytic ascites considered as a less severe variant of SBP [3]. From our study we inferred that if the clinical course, prognosis and mortality rate of culture negative neutrocytic ascites is similar to spontaneous bacterial peritonitis then why should it be considered as a different entity? We argue in favour of calling culture negative neutrocytic ascites as early stage spontaneous bacterial peritonitis. The possibility of late stage resolution of spontaneous bacterial peritonitis leading to culture negativity cannot be ruled out, however the chances are really slim as persons with advanced stage of cirrhosis are too immunocompromised to clear the bacterial infection on its own. May be the bacterial particle in ascitic fluid have not reached the threshold limit to be detected by the current culture methods. We still use the old school method of ascitic fluid polymorphonuclear cell count and culture to diagnose ascitic fluid infections. In my opinion we should incorporate bacterial DNA or pro inflammatory markers to diagnose ascitic fluid infections into our current clinical practice. However, it is critical to access the cost effectiveness and feasibility of such newer methods in developing nations with limited resources. To summarize, the prognostic parameters in culture negative neutrocytic ascites with liver cirrhosis are well known and established for more than a decade now but to my knowledge none of the studies have indicated the predictive ability of alanine and aspartate amiotransferase as far as in-hospital mortality is concerned. In this aspect our study will enlighten the scientific community. Our results showed liver enzymes like aspartate aminotransferase, alanine aminoransaminase have significant prognostic value in detecting mortality of culture negative neutrocytic ascites along with other classic parameters like acute kidney injury, septic shock, model for end stage liver disease.

Our study has certain limitations. We didn’t stratify the septic shock to different sub groups based on bacteriology. The sensitivity and specificity of AST is low (60% and 70% respectively).

## Conclusion

Patients with culture negative neutrocytic ascites have a mortality rate comparable to spontaneous bacterial peritonitis (39.41% vs 43.11%) [8]. Apart from the established predicting factors like acute kidney injury, septic shock and model for end stage liver disease, other clinically relevant factors like aspartate aminotransferase, alanine aminotransferase have significant predictive ability as far as culture negative neutrocytic ascites is concerned. To our knowledge none of the previous studies have mentioned about the prognostic ability of aspartate aminotransferase, alanine aminotransferase in culture negative neutrocytic ascites.

## Abbreviations

AKI: Acute kidney injury
ALT: Alanine aminotransferase
AST: Aspartate aminotransferase
AUC: Area under the curve
CI: Confidence interval
CNNA: Culture-negative neutrocytic ascites
CTP: Child turcotte pugh
HR: Hazard ratio
HRS: Hepato-renal syndrome
INR: International normalized ratio
MELD: Model for end stage liver disease
PMN: Poly-morphonuclear cell
ROC: Receiver operating characteristics
SBP: Spontaneous bacterial peritonitis
